# Non-invasive fundoscopy as a tool to estimate intracranial pressure: a large animal model

**DOI:** 10.1007/s00701-025-06437-3

**Published:** 2025-01-25

**Authors:** Niclas Lynge Eriksen, Frantz Rom Poulsen, Mikkel Schou Andersen, Mathias Just Nortvig

**Affiliations:** 1 Department of Neurosurgery, Odense University Hospital, Odense, Denmark; 2 Department of Clinical Research, Faculty of Health Sciences, University of Southern Denmark, Odense, Denmark; 3 BRIDGE (Brain Research - Interdisciplinary Guided Excellence), University of Southern Denmark, Odense, Denmark

**Keywords:** Intracranial pressure, Non-invasive, Fundoscopy, Arteriovenous ratio

## Abstract

**Purpose:**

Intracranial pressure (ICP) monitoring is in most studies considered essential in avoiding secondary brain injury in patients with intracranial pathologies. Invasive monitoring of ICP is accurate but is unavailable in many clinical and prehospital settings. Non-invasive modalities have historically been difficult to implement clinically. The retinal arteriovenous ratio (A/V ratio) has shown promise through its relationship with ICP.

This study aimed to further elucidate the relationship between ICP, A/V ratio and the intraocular pressure (IOP) measured with non-invasive fundoscopy in a porcine model.

**Methods:**

We achieved controlled values of ICP ranging from normal (5–15 mmHg) to elevated (> 20 mmHg) within the same animal subject. Six pigs were included. ICP and IOP was measured using an intraparenchymal pressure monitor and a tonometer, respectively. Fundoscopy was performed at baseline and at predefined ICP values.

**Results:**

Mixed-effects linear regression revealed a significant inverse correlation between A/V ratio and ICP ≥ 20 mmHg (slope coefficient − 0.0026734 [95%-CI: −0.0039347 – (−0.0014121)], *p* < 0.001). For ICP < 20 mmHg there was no change in A/V ratio (*p* = 0.987). Similar results were seen for ICP > IOP with a mean IOP of 10 mmHg. A Wald test showed no significant difference between ICP > IOP and ICP ≥ 20 mmHg. ROC curve analysis revealed an AUC of 0.64 for ICP ≥ 20 mmHg and 0.71 for ICP > IOP.

**Conclusion:**

The results support the hypothesis that an increase in ICP was associated with a decrease in A/V ratio. Although a slightly better fit, the model of ICP > IOP was deemed less clinically relevant than ICP ≥ 20 mmHg because of the subjects’ IOP.

Further research integrating multifactorial models and machine learning is needed to enhance the diagnostic accuracy of A/V ratio via fundoscopy, enabling it to serve as a cost-effective screening tool.

## Background

The Monro-Kellie doctrine states that total intracranial volume is constant and consists of brain, cerebrospinal fluid (CSF), and intracranial blood [3]. The brain has compensatory mechanisms that allow a suitable and constant milieu through the displaceability of the skull’s contents [5]. This dynamic equilibrium ensures that intracranial pressure (ICP) does not exceed the favorable levels of 5–15 mmHg in adults [9]. If intracranial volume increases beyond the brain’s compensatory capacity, ICP increases, and levels > 22 mmHg usually require treatment to avoid secondary brain injury [6].

Invasive modalities remain the preferred method to measure ICP. They include the use of an intraparenchymal fiber optic probe, lumbar puncture or an external ventricular drain (EVD), with the latter being considered the gold standard [19]. However, the intraparenchymal probe and the EVD carry risks such as hemorrhage or infection [4, 25]. Furthermore, the lumbar puncture is widely used in the diagnosis of neurological conditions such as idiopathic intracranial hypertension, normal pressure hydrocephalus and meningitis. Although the complication rate is lower with lumbar puncture than intracranial modalities, it still carries risks such as headaches, bleeding and infection [8]. As intracranial invasive modalities are only available at highly specialized centers and non are accessible in the prehospital setting, there is a need for non-invasive alternatives to accurately measure ICP. Several non-invasive modalities have been suggested, such as optic nerve sheath diameter or venous ophthalmodynamometry, but none have been clinically implemented due to variable accuracy and limitations in continuous monitoring [13, 19, 23].

The relationship between ocular hemodynamics and acute intracranial hypertension was first described in monkeys [12, 22]. In 2016, an inverse correlation was suggested between the arteriovenous ratio (the A/V ratio) in the fundus of the eye and ICP [2]. The hypothesis was that an increase in ICP would result in inadequate drainage of the retinal veins, dilatation of the retinal veins, and subsequently a decrease in A/V ratio. Furthermore, the intraocular veins have been described to act as Starling resistors in relation to the intraocular pressure (IOP) [24].

A 2020 pilot study found a similar inverse relationship using non-invasive fundus imaging, where potentially dangerous elevations of ICP (≥ 20 mmHg) could be significantly differentiated from non-elevated ICP using the A/V ratio [1]. Hagen et al. recently showed that measuring the A/V ratio could estimate high or low ICP through an inverse relationship with good sensitivity and specificity in patients with idiopathic intracranial hypertension and controls [11]. These findings mandate further research into whether non-invasive fundoscopy can be an alternative to conventional invasive ICP measurement in a clinical setting. An animal model enables a controlled setup for ICP measurement and allows validation across a wide range of ICP values on the same subject.

## Aim and purpose

The aim of this study was to investigate whether the A/V ratio could be correlated with changes in ICP through fundus imaging. By using a porcine model, we were able to control ICP values ranging from normal (5–15 mmHg) to elevated (> 20 mmHg) [20] on the same animal to assess the modality’s ability to distinguish ICP < 20 mmHg from ICP ≥ 20 mmHg as well as ICP ≤ IOP from ICP > IOP. The threshold of 20 mmHg was chosen due to treatment guidelines for intracranial hypertension [6]. Moreover, this study aimed to investigate the variability in baseline A/V ratio, as well as variability in the interaction between ICP and A/V ratio for each animal.

## Methods and materials

This controlled laboratory experiment was performed at the Biomedical Laboratory at the University of Southern Denmark. The study was registered and approved by the Animal Experiments Inspectorate (Journal no. 2023-15-0201–01457) and all methods were in accordance with relevant guidelines and regulations. It was a non-recovery study and ethical considerations for post-surgery care and analgesia was therefore not relevant. The study complied with the ARRIVE 2.0 guidelines.

### Experimental animals

Six female pigs of the strain LY obtained from Kokkenborg (Stenstrup, Denmark) were included. The pigs arrived simultaneously at the Biomedical Laboratory and acclimatized for at least one week before the start of experiments. They were 14.5 weeks old, and their mean weight was 41.3 Kg (SD: 1.7) (Table 1).

**Table 1:** Baseline data of animal subjects

Pig no.	Weight (Kg)	Median baseline A/V ratio	Baseline ICP (mmHg)	Baseline ABP* (mmHg)	Maximal ICP ABP* (mmHg)	IOP† (mmHg)
1	39.3	0.85	7	126/52	179/105	11/9
2	40.1	0.87	5	118/48	114/50	12/15
3	40.9	0.69	11	101/81	150/73	17/10
4	43.3	0.87	10	108/57	122/71	13/10
5	43.9	0.81	9	112/56	85/63	5/4
6	40.3	0.72	7	114/52	123/72	5/9

*Arterial blood pressure (ABP) given as systolic/diastolic

†IOP given as right eye/left eye

### Anesthesia and euthanasia

Anesthesia was induced with an initial dose of 7.5 mL of a balanced anesthetic containing medetomidine, ketamine, butorphanol, and midazolam (CP Pharma, Germany) administered as an intramuscular injection. After oral intubation, the pigs were given weight-adjusted intravenous (IV) infusions of propofol and fentanyl to maintain anesthesia. The drugs were co-infused with Ringer’s Acetate (Fresenius Kabi, Sweden) at 160 mL/h. After data collection, the pigs were euthanized using a weight adjusted IV bolus of pentobarbital (Vetviva Richter, Austria).

### Experimental procedure

**Fig. 1:**
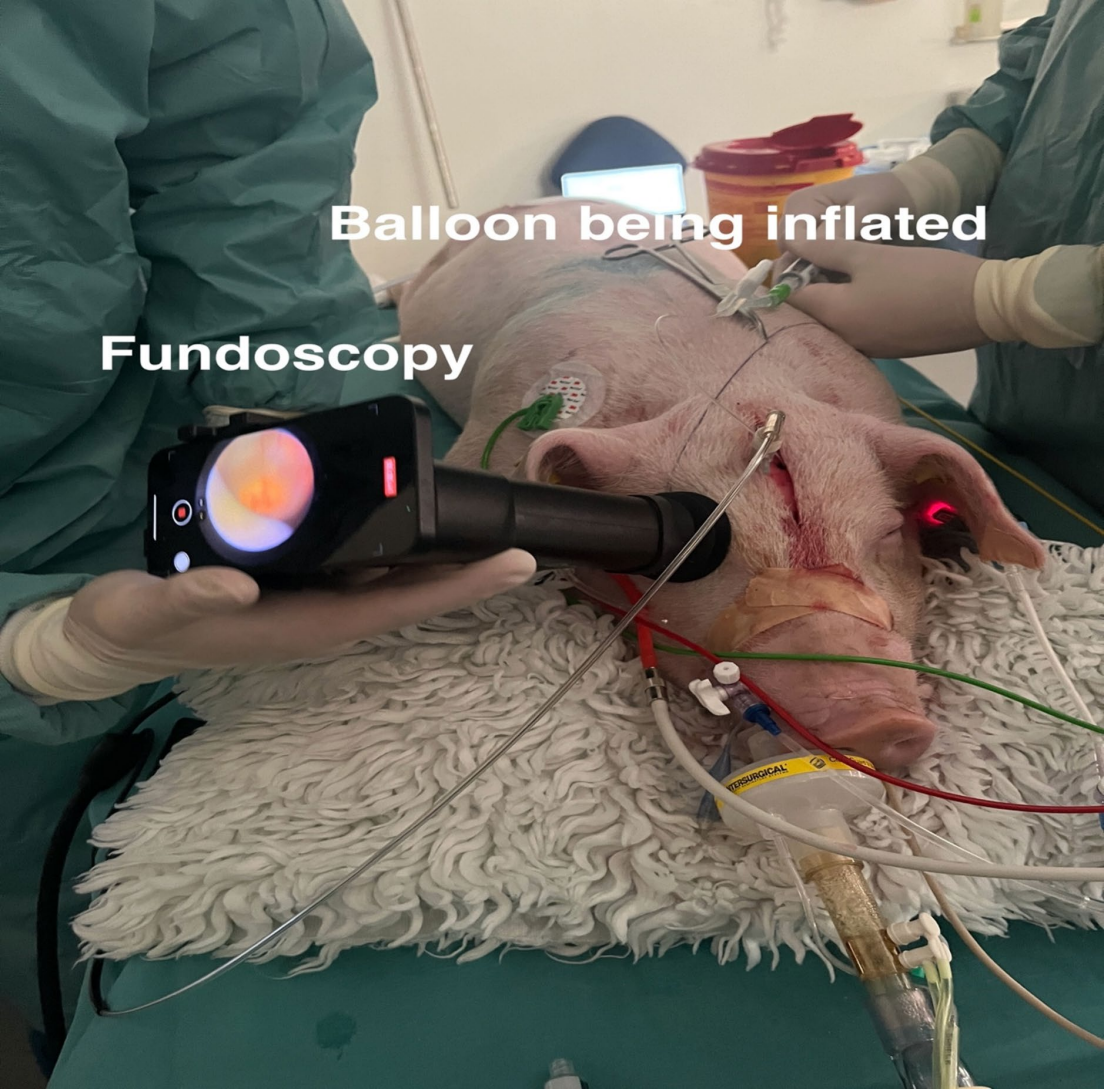
Experimental setup showing the two frontal burr holes. On the pig’s right, an intraparenchymal pressure monitor. On the pig’s left, an epidurally placed catheter with a balloon being inflated to specific ICP values

The pigs were placed in prone position. Using a scalpel, a midline incision was made on the frontal part of the skull. The periosteum was pushed aside using a periosteal elevator to reveal the skull. Two burr holes were placed just anteriorly of the coronal suture, one on each side of the midline. All ICP measurements were captured using the intraparenchymal fiber optic probe system Camino^®^ Intracranial Pressure Catheter 1104BT (Natus, Middleton, USA) and monitor (Integra NeuroCare LLC San Diego, USA). The probe was calibrated and placed according to the manufacturer’s guidelines. It was placed on the right side. On the left side, a size 6 bladder catheter was inserted in the burr hole epidurally (Fig. 1). MJN raised the ICP by slowly (over 20 s) inflating the catheter balloon with saline until the first desired ICP level. When ICP was stable at the desired level, fundoscopy was performed. ICP was then slowly increased in increments of 5 mmHg and fundoscopy was performed again. This stepwise approach resulted in no sudden ICP spikes. If the balloon burst at an ICP around 25 mmHg, it was replaced by a new catheter (size 8) until the end of data collection.

We continuously monitored and recorded the pig’s heart rate, peripheral oxygen, end tidal CO_2_, ICP, and arterial blood pressure using a webcam pointed at the monitors. Arterial blood pressure was obtained by cannulation of the femoral artery and used as a pseudomeasure for compensatory functions. Video-fundoscopy was performed by NLE using the Onun Ophthalmoscope (Odocs Eye Care, Dunedin, New Zealand). Fundoscopy was performed at baseline before insertion of the catheter and as ICP was gradually increased. IOP was measured by NLE at baseline with the ICare IC100 tonometer. After sedation and during the procedure, the pigs were monitored by a veterinarian. At any sign of stress, the procedure would have been stopped.

### Image processing

**Fig. 2:**
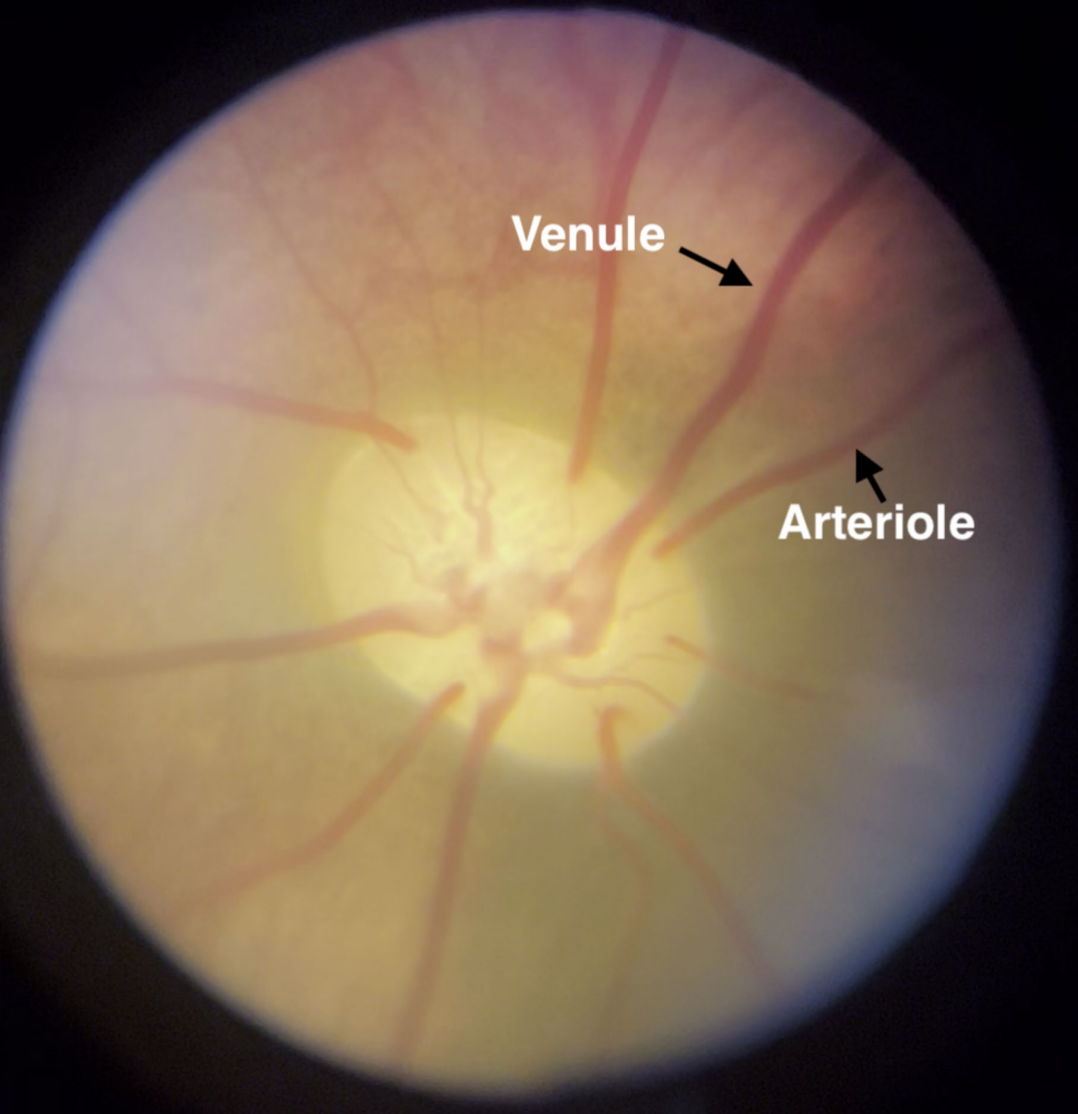
Optic disc and vessels. Vessels are visually distinguishable as the venules are larger and redder while arterioles have a more distinct central reflex

Each fundoscopy video was split into its individual frames. All frames were manually studied, and the five highest quality frames per video were selected. If a video did not have five suitable frames, fewer frames were selected. Figure 2 shows a suitable frame for manual study. The selected frames were randomized and blinded by an independent third party with no relation to the study to minimize observer bias. The randomized, blinded frames were imported to a Python program for manual marking of retinal arterioles and venules. The superior arteriole and venule pair were identified. In accordance with consensus from published studies, the superior vessel diameters were measured at approximately two optic disc radii from the perimeter of the optic disc [1, 11, 21]. The vessel diameters were measured by two independent observers at five measurement points per frame and a median value was calculated. The diameter was defined as the outer diameter of the vessel (i.e. including the vessel wall). The accepted interobserver variability on the median diameter was 20%, otherwise the images would be reevaluated by both observers. The arteriole and venule diameters were then averaged between the two observers and the A/V ratio was calculated. ICP values were extrapolated from the webcam videos and correlated to the frames according to time stamps. The mean ICP was calculated from the ICP values 10 s before and after the time stamp of the fundoscopy videos. The videos were then unblinded and the A/V ratio was correlated to the mean ICP values.

### Statistics

All statistics were performed in Stata 18 (StataCorp, Texas, USA). Mixed-effects linear regression was used to compare the coefficient of the A/V ratios for the ICP groups < 20 mmHg and ≥ 20 mmHg with A/V ratio being the dependent variable, ICP as the independent and the individual animal subjects as a random effect. A Wald test was performed to compare the slopes of ICP > IOP and ICP ≥ 20 mmHg. Sensitivity analysis was performed on ICP groups < 20 mmHg and ≥ 20 mmHg, and a ROC-curve was created.

## Results

**Fig. 3:**
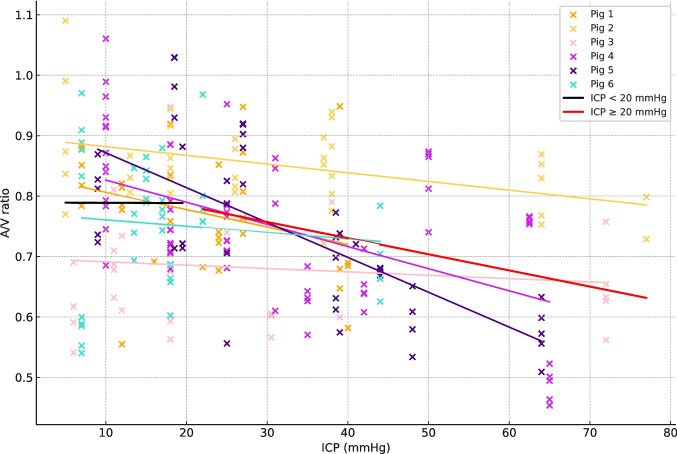
Plot based on 243 observations with mean ICP on the X-axis and A/V ratio on the Y-axis. Each trendline represents the change in A/V ratio for its respective animal. The black trendline represents the summarized change in A/V ratio for ICP < 20 mmHg. The red trendline represents the summarized change in A/V ratio for ICP ≥ 20 mmHg

Mixed-effects linear regression was used to assess the relationship between A/V ratio and ICP values < 20 mmHg or ≥ 20 mmHg. Mean ICP ≥ 20 mmHg showed a significant inverse correlation with A/V ratio with a slope coefficient of −0.0026734 [95%-CI: −0.0039347 – (−0.0014121)], *p* < 0.001. For mean ICP < 20 mmHg, there was a non-significant inverse correlation between A/V ratio and ICP with a slope coefficient of −0.0000357 [95%-CI: −0.0044996–0.0044282], *p* = 0.987. This suggests that the A/V ratio remains stable until ≈ 20 mmHg whereafter the A/V ratio starts decreasing as ICP increases as seen in Fig. 3.

Two-way analysis of variance (ANOVA) was used to assess whether the animal subjects showed variance in their A/V ratio and in their response to changes in ICP. The analysis for individual animals gave an F-statistic of 10.66 and a P-value < 0.01, meaning that each animal had a significantly different baseline A/V ratio and the between-group variance was therefore 10.66 times larger than the within-group variance [14]. The interaction effect of ICP on A/V ratio, also assessed using ANOVA, gave an F-statistic of 3.01 and a P-value of 0.0119.

**Fig. 4:**
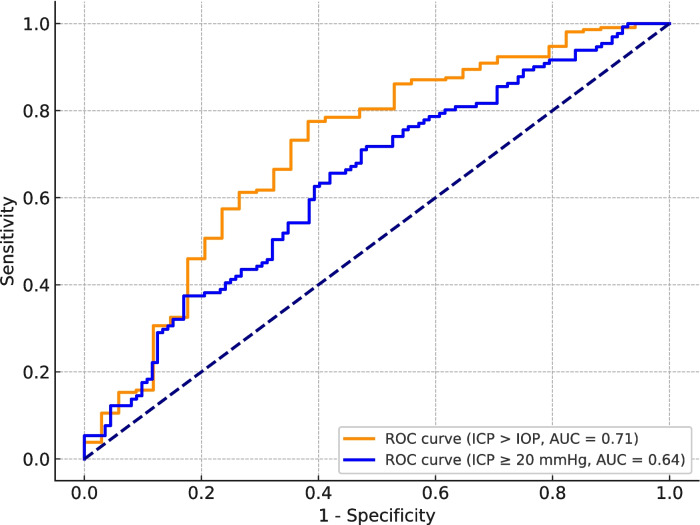
ROC curve comparison. Orange curve depicts the A/V ratio’s ability to classify ICP > IOP. The blue curve depicts the A/V-ratio’s ability to classify ICP ≥ 20 mmHg

The ability of the A/V ratio to correctly classify ICP ≥ 20 mmHg and < 20 mmHg was assessed using receiver operating characteristic (ROC) curves and sensitivity analyses. This showed an area under the curve (AUC) of 0.64, shown in Fig. 4. Sensitivity analysis showed a sensitivity of 80.92% and a specificity of 36.61% with a cut-off value at 0.83. The positive predictive value was 53.91% and the negative predictive value 72.55%, meaning that 87.5% of cases with an ICP ≥ 20 mmHg would be classified as positive. However, it also resulted in a substantial number of false positives due to the low specificity of 36.61%.

The IOP ranged from 4 mmHg to 17 mmHg (Mean: 10 mmHg). The data were therefore analyzed with IOP as variable as well. For mean ICP > IOP, there was a significant inverse correlation with A/V ratio with a slope coefficient of −0.0022034 [95%-CI: −0.0030737 – (−0.0013331)], *p* < 0.001. For mean ICP ≤ IOP, there was a non-significant inverse correlation between A/V ratio and ICP with a slope coefficient of − 0.0121835 [95%-CI: −0.0314743–0.0071074], *p* = 0.216. ROC curve analysis for ICP > IOP revealed an AUC of 0.71. This resulted in a sensitivity of 86.12% and a specificity of 47.06% at a A/V ratio cut-off point at 0.87.

A Wald comparison of slopes between ICP > IOP and ICP ≥ 20 mmHg showed a Z-score of 0.60 and a *p*-value = 0.55. This means that there was no significant difference between the two slopes, and IOP therefore did not add significantly to the results.

## Discussion

This is the first study to investigate the relationship between ICP, IOP and A/V ratio with controlled values of ICP in a porcine model. The results support the hypothesis that an increase in ICP is associated with a decrease in A/V ratio as previously suggested [1, 11, 17]. The mechanism for ocular hemodynamics was observed to be primarily driven by the distension of the venule in accordance with earlier findings [1, 11, 16]. The driving factor behind the distension of the venule is likely to be the change in translaminar pressure difference (TLPD) with increasing ICP. This mandates further studies into hemodynamic parameters such as TLPD, ocular perfusion pressure and episcleral venous pressure to refine the model and further the predictive values of the A/V ratio.

Since the retinal vein flow seems dependent on IOP [15, 24], we compared a clinical cut-off of ICP ≥ 20 mmHg with a physiological cut of ICP > IOP. A/V ratio’s ability to classify ICP > IOP showed a slightly better AUC (0.71 vs. 0.64). However, when trying to assess absolute ICP from a single measurement, ≥ 20 mmHg seems more clinically applicable. Moreover, this would also simplify the clinical test by not relying on the availability of a tonometer that may have varying accuracy.

The results support the conclusion of Andersen et al., 2020 that the A/V ratio can be used to detect relative changes in ICP because of the significant difference in slope coefficients for normal and elevated ICP [1]. Our Wald test showed no significant difference in slopes for ICP ≥ 20 mmHg and ICP > IOP.

The A/V ratio demonstrated a high sensitivity of 80.92% but a low specificity of 36.61%, leading to a considerable number of false positives in predicting ICP ≥ 20 mmHg. Therefore, this non-invasive approach may be best suited to monitor relative changes in ICP rather than detecting intracranial hypertension from a singular measurement. This could for example be for patients that suffered from an intracranial hemorrhage and are being observed on periphery hospitals where invasive neuromonitoring is not available. These patients would greatly benefit from a non-invasive modality that could detect changes in ICP and possible prognosticate the need for intervention.

Significant variation was observed in the pigs’ baseline A/V ratios despite their similarities in gender, age, and weight. This variation likely contributed to the low specificity, consistent with similar findings reported in humans [1, 11]. Specifically, pig no. 3 exhibited a low baseline A/V ratio and a smaller change in A/V ratio than the others, which greatly affected the sensitivity analysis. Individual variability in baseline A/V ratio is thus likely, but it remains unclear whether this is entirely related to individual variability or was influenced in the current study by artifacts related to the fundus camera quality. The two-way ANOVA strongly suggested physiological individual variability (*p* < 0.001), but the interaction effect was less pronounced (*p* = 0.012). We hypothesize that better camera quality would reduce the F-statistics for the interaction effect but would have lower impact on the individual variability in baseline A/V ratio. This would imply that relative changes in A/V ratios across subjects would remain comparable when ICP increased while the baseline A/V ratios would reflect individual variability.

Our findings suggest that non-invasive fundoscopy currently cannot replace invasive modalities but could serve as a tool to detect relative changes in ICP in setting where invasive monitoring is contraindicated or unavailable.

A strength of the current animal study is the controlled increase in ICP that enabled measurement of the A/V ratio across a wide range of ICP values on the same animal subject, including values above 20 mmHg. Treatment guidelines for acute intracranial hypertension mean that most datapoints from human studies investigating the relationship between ICP and A/V ratio fall within the range of 0–20 mmHg. Our data suggest that within this range, there would be no significant change in the A/V ratio. Moreover, the model is based on a mammal with a large brain that can handle ICP monitoring probes and has physiology that borders humans [18]. Another strength is that the fundoscopic frames were randomized and blinded by an independent third party, and the vessel diameters were measured by two independent observers. This minimized the risk of observer bias.

A limitation of our study is the innate physiological differences between pigs and humans, which could affect the external validity. Furthermore, we were able to include six pigs only. A larger cohort would have been optimal. Finally, because this study was a none-recovery study, it was not possible to perform multiple measuring sessions on the same animals.

A/V ratio, in its simplicity, has shown a correlation with ICP but currently lacks the ability to determine ICP from a single measurement. Therefore, it is necessary to incorporate multiple parameters obtained from fundoscopy, such as spontaneous venous pulsation or peripapillary changes [7, 10]. A multifactorial model might be able to estimate an absolute value of ICP from a single measurement by incorporating enough variables to account for individual variability. However, this would currently require higher quality cameras that could allow evaluation of these parameters. Finally, technological advances in artificial intelligence and machine learning may fully automate the process, eliminating inter- and intra-observer variability.

## Conclusion

In this study, non-invasive fundoscopy was investigated as an easily available tool for estimating ICP. A significant difference in A/V ratio was observed between ICP < 20 mmHg and ICP ≥ 20 mmHg (*p* < 0.001). This was also observed for ICP > IOP (*p* < 0.001). However, because of a mean IOP of 10 mmHg this correlation was deemed less clinically relevant despite being a slightly better fit. These findings align with earlier research demonstrating the ability of A/V ratio measured through fundoscopy to detect relative changes in ICP. Because of the low specificity and high sensitivity of this approach, A/V ratio would appear to have potential as a tool of detecting relative changes in ICP rather than an absolute value from a singular measurement. Further development of multifactorial models of A/V ratio and relevant variables, as well as machine learning, would help to progress the predictive value of fundoscopy as an easily available modality to accurately estimate ICP. These models should be validated in large, human cohorts.

## Data Availability

All data and materials are available upon contact to the corresponding author.

## Abbreviations

ICP: Intracranial pressure
IOP: Intraocular pressure
A/V ratio: Arteriovenous ratio
CSF: Cerebrospinal fluid
EVD: External ventricular drain
IV: Intravenous
ANOVA: Analysis of variance
ROC: Receiver operating characteristic
AUC: Area under the curve
TLPD: Translaminar pressure difference with increasing ICP
